# Health care for immigrants in Europe: Is there still consensus among country experts about principles of good practice? A Delphi study

**DOI:** 10.1186/1471-2458-11-699

**Published:** 2011-09-13

**Authors:** Walter Devillé, Tim Greacen, Marija Bogic, Marie Dauvrin, Sónia Dias, Andrea Gaddini, Natasja Koitzsch Jensen, Christina Karamanidou, Ulrike Kluge, Ritva Mertaniemi, Rosa Puigpinós i Riera, Attila Sárváry, Joaquim JF Soares, Mindaugas Stankunas, Christa Straßmayr, Marta Welbel, Stefan Priebe

**Affiliations:** 1 International and Migrant Health, NIVEL (Netherlands Institute for Health Services Research), Otterstraat 118-124, PO Box 1568, 3500 BN Utrecht & University of Amsterdam, Amsterdam Institute of Social Sciences Research, Amsterdam, the Netherlands; 2 Etablissement public de santé Maison Blanche, 18 rue Rémy de Gourmont, 75019 Paris, France; 3 Unit for Social and Community Psychiatry, Queen Mary University of London, Newham Centre for Mental Health, London, E13 8SP, UK; 4 Institute of Health and Society, Université Catholique de Louvain, Clos Chapelle aux Champs 30.05., 1200 Brussels, Belgium; 5 Instituto de Higiene e Medicina Tropical, Universidade Nova de Lisboa, Rua da Junqueira, 96, 1349-008 Lisbon, Portugal; 6 Laziosanità ASP-Public Health Agency for the Lazio Region, Via S. Costanza 53, 00198 Rome, Italy; 7 Danish Research Centre for Migration, Ethnicity and Health (MESU), Section for Health Services Research, Department of Public Health, University of Copenhagen, Øster Farimagsgade 5, DK-1014 Copenhagen, Denmark; 8 Department of Sociology, National School of Public Health, 196 Alexandras Avenue, Athens 11521, Greece; 9 Clinic for Psychiatry and Psychotherapy, Charité-University Medicine Berlin, CCM, Charitéplatz 1, 10117 Berlin, Germany; 10 National Institute for Health and Welfare (THL), Department for Mental Health and Substance Abuse Services, P.O.B. 30, FIN-00271 Helsinki, Finland; 11 Agency of Public Health of Barcelona, Pça. Lesseps, 1, 08023 Barcelona, Spain; 12 Faculty of Health Sciences at Nyíregyháza, University of Debrecen, Sóstói út 31/B, 4400 Nyíregyháza, Hungary; 13 Department of Public Health Sciences, Section of Social Medicine, Karolinska Institutet, SE-171 76 Stockholm, and Department of Public Health Sciences, Mid Sweden University, SE-851 70 Sundsvall, Sweden; 14 Department of Health Management, Lithuanian University of Health Sciences, A. Mickevičiaus g. 9, LT 44307, Kaunas, Lithuania; 15 Ludwig Boltzmann Institute for Social Psychiatry, Lazarettgasse 14A-912, 1090 Vienna, Austria; 16 Institute of Psychiatry and Neurology, Ul. Sobieskiego 9, 02-957 Warsaw, Poland

## Abstract

**Background:**

European Member States are facing a challenge to provide accessible and effective health care services for immigrants. It remains unclear how best to achieve this and what characterises good practice in increasingly multicultural societies across Europe. This study assessed the views and values of professionals working in different health care contexts and in different European countries as to what constitutes good practice in health care for immigrants.

**Methods:**

A total of 134 experts in 16 EU Member States participated in a three-round Delphi process. The experts represented four different fields: academia, Non-Governmental Organisations, policy-making and health care practice. For each country, the process aimed to produce a national consensus list of the most important factors characterising good practice in health care for migrants.

**Results:**

The scoring procedures resulted in 10 to 16 factors being identified as the most important for each participating country. All 186 factors were aggregated into 9 themes: (1) easy and equal access to health care, (2) empowerment of migrants, (3) culturally sensitive health care services, (4) quality of care, (5) patient/health care provider communication, (6) respect towards migrants, (7) networking in and outside health services, (8) targeted outreach activities, and (9) availability of data about specificities in migrant health care and prevention. Although local political debate, level of immigration and the nature of local health care systems influenced the selection and rating of factors within each country, there was a broad European consensus on most factors. Yet, discordance remained both within countries, e.g. on the need for prioritising cultural differences, and between countries, e.g. on the need for more consistent governance of health care services for immigrants.

**Conclusions:**

Experts across Europe asserted the right to culturally sensitive health care for all immigrants. There is a broad consensus among experts about the major principles of good practice that need to be implemented across Europe. However, there also is some disagreement both within and between countries on specific issues that require further research and debate.

## Background

The globalization of migration flows, over recent decades, has increased the multicultural diversity of our societies. Globally, the annual flow of immigrants between 2005 and 2010 was estimated to be around 2.7 million, with about 100 million migrant workers in 2009 [1]. According to the Organisation for Economic Co-operation and Development (OECD), the percentage of the foreign-born population within the European Community in 2008 ranged from 4% in Finland to 37% in Luxembourg, with an overall average of 8% [2,3].

Health care services in these countries have to deal with increasingly culturally diverse populations. The World Health Organisation (WHO) mentioned in its Fact Sheet n° 31 "*The Right to Health*" that the right to health implies equal and timely access to health care services, the provision of health-related education and information, and the participation of the population in health-related decisions at national and community levels [4]. The right to health also implies equity in access to health care services for equal health needs. Health care services should be physically and financially accessible for all sectors of the population, including vulnerable groups, and should be delivered on the basis of non-discrimination. Facilities, goods, and services should respect medical ethics, as well as being gender-sensitive and culturally appropriate. In other words, they should be culturally and not only medically acceptable. Finally, they must be scientifically appropriate and of good quality. In short, access to health care is more than a legal right to health care. Many other aspects are involved.

Meeting the health needs of migrants and ethnic minorities is a challenge for health care services. Inequitable variation in the use and accessibility of health care services for migrants, indigenous populations, and other minorities in EU countries is a matter of concern for both health care providers and policy-makers. A substantial body of scientific literature [5-12] and policy reports [13-17] have documented differences between migrant groups and local populations in health care utilization in Europe. Migrants do not always have the right to the services they require (e.g. due to their legal status). Even in countries where access to health care is guaranteed for all migrant populations, they often meet with obstacles to quality care for: individual, socio-cultural, economic, administrative and political reasons [18,19]. There is therefore mounting pressure at the European level to ensure equal access for migrants to social and health care services, as reflected in the Portuguese Presidency of the EU in 2007 for example [20-22]. Governments are increasingly encouraged to develop policies to reduce migrant health care inequalities [23].

The thrust towards harmonisation across the EU makes the generation of evidence on what constitutes good practice in health care for migrants across Europe timely, with a view towards wider dissemination and implementation. The history and level of immigration vary between Member States, resulting in different levels of experience in providing health care to culturally diverse populations. In countries where such data is available, migrants seem to make lower use of specialist inpatient and outpatient care, with greater utilisation of emergency services instead [24]. Although barriers to accessing and using health care services have been described in several countries [25], good practices for dealing with these barriers have not yet been researched in any systematic way across Europe. There has been even less systematic research on how health care should best be delivered, once an immigrant has entered a service. The current study aimed to assess the views and values of professionals working in different health care contexts in 16 European countries as to what constitutes good practice in health care for immigrants.

## Methods

The study is part of the project "*Best Practice in Access, Quality and Appropriateness of Health Services for Immigrants in Europe" *(EUGATE). Funded by the European Commission, the project collected data in 16 Member States: Austria, Belgium, Denmark, England, Finland, France, Germany, Greece, Hungary, Italy, Lithuania, Netherlands, Poland, Portugal, Spain and Sweden. Details and other findings from the project have been described elsewhere [26].

The present article describes results from another part of the project, a Delphi process on principles of good practice with experts from all 16 countries.

The Delphi method was developed as a means for collecting and synthesizing expert opinion on a given issue in the area of their expertise. Participants are chosen for their expertise, and are ensured anonymity with respect to their opinions. In light of the replies of other participants, they were then encouraged to revise their initial answers. The process aimed to bring the group towards a consensus. The process also produced a set of arguments defending the different points of view, whether they were consensual or discordant [27]. Three rounds are commonly viewed as sufficient for arriving at a high level of agreement [28]. For strengths and limitations of the Delphi method, see Hung et al. [28].

In order to identify principles of good practice, eight experts in the field of migration and health were recruited in each of the 16 participating countries (11 in Belgium and Germany). Experts were chosen primarily for their experience and expertise in the area of health care for migrants. In each country, experts were purposively recruited to ensure representation from each of four different backgrounds: academia, the non-governmental sector, policy-making and health care practice (Table 1). No other criteria were set for recruitment.

**Table 1:** Background of participating experts at the beginning of the Delphi Process in each country

COUNTRY	Academia	NGO	Policymaker	Practitioner
AUSTRIA	Sociology (3)	Political sciences	Political sciences (2)	Medicine (2)
BELGIUM	Public health Sociology (2) Pedagogy	Medicine Social work	Medicine Anthropology	Public health Medicine (2)
DENMARK	Psychiatry(2)	Psychiatry	Public Health Sociologist	Public health Internal medicine Medicine
ENGLAND	Researcher Nursing	Social work	Medicine (2)	Psychiatry Occupational therapy Psychology
FINLAND	Social sciences Master of Arts in Comparative Religion	Psychiatric nursing Psychiatry	Law	Psychiatry Public health Nursing (2)
FRANCE	Public health (2)	Medicine Social work	Public health Psychologist	Psychiatry Medicine
GERMANY	Psychiatry (3) Political sciences Sociology Medical psychology	Psychotraumatology Political sciences NGO activist	Social sciences	Psychiatry
GREECE	Law Public health	Medicine	Health economy	Psychiatry Psychology Medicine (2)
HUNGARY	Sociology Geography	Economical sciences Chemical sciences	Social worker (2)	Medicine (2)
ITALY	Public health Psychiatry	Psychiatry Medicine	Psychiatry (2)	Public health Psychiatry
LITHUANIA	Medicine Medical anthropology	Medicine Social Sciences/Law	Public health (2)	Gynaecology Medicine
NETHERLANDS	Sociology Epidemiology	Paediatrics Medical anthropology	Management Public Health	Medicine (2)
POLAND	Political sciences Migration studies	Cultural anthropology NGO Activist	Law Psychology	Medicine (2)
PORTUGAL	Medicine Sociology	Medicine Biochemistry	Intercultural relations Nursing	Public Health Nursing
SPAIN	Human Geography Economical Sciences	Medicine (2)	Medicine (2)	Gynaecology Psychology
SWEDEN	Public Health Psychology	Nursing	Nursing Sociology Psychology	Medicine (2)

From June 2008 to January 2009 data were collected either: (a) a specifically created online software programme in seven countries or (b), where researchers considered that the use of English-language software risked compromising participation for certain experts, via e-mail correspondence directly with experts. The Delphi process was divided into four rounds following a standard protocol. The experts were first invited to suggest up to ten statements describing factors that in their opinion constituted "best practice in the delivery of health care services to migrants". They were asked to focus on health care delivery, from their perspective, for migrants who: (a) had arrived in their country within the last five years, (b) were between 18 and 65 years of age, (c) had a regular legal income, and (d) did not originate from a developed country with a similar language (e.g. a Canadian from Quebec moving to Belgium). For each factor, they were asked to provide a brief explanation as to why they considered this factor to be important.

The total list of initial factors in each country was then reviewed by a minimum of two researchers in each local research team using the following instructions: (i) factors that were identical or using only slightly different wording to describe the same phenomenon were grouped into one factor; (ii) factors covering more than one phenomenon were split into more discreet entities. Each research team sought to respect the nuances given to the factors by the experts, using the provided explanations and aiming to define factors that would be meaningful for the same experts during the second round. At the end of this process, each individual factor consisted of a single summary statement, followed by an explanation composed by the research team but using only the comments provided by the experts themselves.

In the second round of the Delphi process, the revised list of factors, each with its explanation, was sent back to the experts who were asked to rate the importance of each factor on a scale from 1 (not important) to 5 (very important). An average score based on the scores of the individual experts was calculated for each factor. In the third round, experts whose ratings varied by more than one point from the average group rating (rounded off) on any particular factor, were asked to reconsider their rating on that factor in the light of the average score for all experts in their country. It was specifically mentioned that they had no obligation to revise their score, but, if they chose not to revise it, they were asked to explain their position. Ratings differing by less than one point from the rounded average score were regarded as consensus. For the final data analysis, individual discordance within countries was defined as an expert giving a final third round score that differed by 3 points or more from the rounded average score for that country. Between countries, discordance was defined as the presence of a good practice factor in a final national list that was in contradiction with a good practice factor in the final list of another country. Discordant experts explained why they remained with their scores.

In the presence of persisting within-country discordance, only one country organized a fourth round. This was due to the reticence of certain experts to modify their discordant scores, regardless of whether this meant that the factor in question would no longer be in the top ten for that country. Other countries did not organize a fourth round. After the last round, the factors with the ten highest average scores in each country were selected as the final list for that country. In countries where several factors scored identical for the tenth position, more than ten were included in the final list. Research teams in each country discussed the outcomes, considering both the national context at the time of the data collection, and any potential reasons for discordance. For the final analysis, all national good practice lists were translated into English where necessary. The final national lists were then compared to assess the level of consensus across Europe. Issues of discordance within and between countries were also reviewed.

## Results

Of the 134 experts in the 16 countries who were recruited into the Delphi process, 126 completed the last round. The eight experts who dropped out did so after the second round. Data on participating countries and the professional background of the experts is presented in Table 1.

The local reviewing process to remove repetition at each site prior to round two reduced these lists to between 11 and 40 factors for each country.

Final national consensus lists after the third round included between 10 and 16 factors with the ten highest scores, resulting in a total of 186 high scoring factors across all countries (Table 2).

**Table 2:** Number of factors of good practice listed per Delphi round and range of final scores

COUNTRY	1st round	2nd round	Final	Scores
AUSTRIA	48	17	11	4.4-4.0
BELGIUM	91	21	10	4.6-4.3
DENMARK	60	32	11	5.0-4.4
ENGLAND	64	28	10	4.9-3.5
FINLAND	50	22	13	4.9-4.0
FRANCE	84	41	16	4.9-4.1
GERMANY	64	30	12	4.8-4.2
GREECE	24	24	10	4.6-4.0
HUNGARY	65	16	10	4.0-3.3
ITALY	80	11	10	4.6-3.8
LITHUANIA	45	35	14	4.8-4.1
NETHERLANDS	54	26	12	4.9-3.9
POLAND	63	31	16	4.8-4.0
PORTUGAL	57	18	10	4.8-4.0
SPAIN	75	14	10	5.0-3.2
SWEDEN	76	30	11	4.9-4.5

### Consensus

The 186 factors fall into nine thematic categories, which are summarized below in order of frequency, and are detailed further in Table 3. The first eight themes figured in the final national good practice lists for more than half of the participating countries. The themes represent general principles, some generic but with specific aspects for migrants. The nine general principles were:

**Table 3:** Major themes in the 16 country-specific, final factor lists

Theme	Description	Details	Countries mentioning theme	Countries not mentioning theme in first 10
Easy and equal access	A health care system that is easy to access for migrants	• Accessibility on the same terms as the general population:	16	
		◦ *In health care systems with free access*, free access to care for migrants.		
		◦ *In insurance-based health care systems*, migrants should have the right to be insured.		
		• Remove barriers to accessing *secondary care*.		
		• *Special health services *should be made available in areas with high migrant populations		
		• *Service hours *should adapt to the needs of their users, including migrants.		
		• Services should be *affordable*: governments should provide the necessary resources and adapt legislation to achieve this.		
		• *Inform health professionals *about the *legislation *related to *the rights to health care *for migrants in their country.		

Empowerment	Empower migrants with regard to health & health determinants	• Provision of *information *for migrants in their *own language*	15	DK
		◦ about their *rights *and the functioning of the *health care system and social care system*.		
		◦ about *health, illness *and *prevention*		
		◦ Provide a *special consultation *the first time people access the health care system.		
		• Outside the health care sector:		
		◦ improving access to work as well as work and living conditions empowers migrants and may consequently		
		◦ improve their health. providing opportunities to learn the language of the host country will facilitate integration into the host country and consequently also access to health care.		
		• *Participation of migrants and non-governmental organisations *(NGOs) dealing with migrants in the organisation of health care services.		

Culturally sensitive care	Adjust care provision to cultural differences	• Health care providers should receive *specific training on cultural competencies and communication skills*.	14	FI, UK
		• Employ *cultural mediators *or *health care providers of migrant descent*,		
		• Develop *specialised services *in case of added value to regular services can be demonstrated.		
		• *Health education and health promotion *messages should take into account cultural diversity.		

Quality care	Guarantee quality of care	• Services should consider *the patient as an individual *and not stereotype them with the characteristics of the cultural group they are perceived of as belonging to.	12	DE, LT, PT, UK
		• Quality care means taking into account *the individual's specific medical history and social background *and giving individualised psychological support and empathy.		
		• Health care professionals should take *the time to listen to patients *and check that both parties have understood each other.		
		• Other factors mentioned:		
				
		◦ establishing *trust*,		
		◦ seeking *truly informed consent*,		
		◦ guaranteeing *continuity of care*		
		◦ *adapting care *to the person's lifestyle and their capacity to receive and self-manage care		

Patient-health care provider communication	Provide interpreting and translation	• High quality *interpreter services*, either in person or by telephone, should be easily accessible.	11	BE, HU, IT, NL, PT
		• Services should take into account varying levels of *both health literacy and mastery of the local language*.		

Respect towards migrants	Fight discrimination & prejudice, respect differences	• *Practitioners *should show *respect*, create *trust*, be *interested *and address patients without prejudice and with an open mind.	9	AT, BE, DE, EL, IT, PT, UK
		• *Health care services *should be delivered without xenophobia or any sign of racism.		
		• *Health care providers *should be motivated to deliver care for migrants with attention to their *specific needs and priorities*.		
		• A *policy against acts of discrimination in health care *facilities should be established and implemented.		

Networking in and outside health care services	Effective networking, integrated care	• *Networking *within health care services and *between health and social services*	8	AT, BE, DE, EL, FR, HU, LT, SE
		• *Interdisciplinarity *is a priority within health care services.		
		• *Coordination *between primary care services, or between primary care and refugee-specific health care services.		
		• *Supporting *migrants to develop their *social networks*		
		• *Supporting *migrants or persons of migrant descent *who care for other migrants*.		

Targeted outreach activities	Targeted outreach programmes in prevention and care	• *Outreach activities *in health education, screening, prevention and promotion with difficult to reach migrant groups.	8	AT, BE, DK, FI, LT, NL, PL, SE

Availability of data	Data on migrants, epidemiology, research	• *Health care services *should be provided with *relevant knowledge on health and risk factors *concerning the populations they are dealing with.	6	DK, EL, FR, HU, IT, LT, PL, PT, SE, UK
		• *Health registries*		
				
		◦ should record and monitor migrant health to facilitate migrant health research.		
		◦ should be able to integrate patient mobility with full respect of human rights.		

• *Accessibility: easy and equal access to health care (mentioned by all 16 countries)*

This theme was mentioned on consensus lists of all countries as the number one priority. All countries mentioned the need for an easily accessible general health care system for all citizens (Table 3). Although they had initially been asked to propose good practice principles for migrants with regular, legal incomes and speaking the local language, experts from several countries also specifically prioritised equal access for refugees and undocumented migrants.

• *Empowerment of migrants (15 countries)*

Migrants should be informed about their rights and the functioning of the health care system. This might involve a special consultation the first time people access the health care system. (Table 3)

• *Culturally sensitive health care (14 countries)*

Optimising culturally sensitive care several specific measures were proposed. (Table 3) Although most countries' experts underlined the need for health care services to take into account the cultural or religious habits of migrants, others considered that migrants should be encouraged to understand the habits and culture of local health care systems.

• *Quality of individual care (12 countries)*

Services should consider the patient as an individual and not stereotype them with the characteristics of the cultural group they are perceived of as belonging to. (Table 3)

• *Patient-health care provider communication (11 countries)*

High quality interpreter services, either in person or by telephone, should be easily accessible. (Table 3).

• *Respect towards migrants (9 countries)*

Practitioners should show respect, create trust, be interested and address patients without prejudice and with an open mind. (Table 3)

• *Networking and interdisciplinarity (8 countries)*

Meeting the health care needs of migrants requires networking within health care services and between health and social services. (Table 3)

• *Targeted outreach activities (8 countries)*

Outreach activities in health education, screening, prevention and promotion with difficult to reach migrant groups were mentioned in eight countries.

• *Availability of data (6 countries)*

Health care services should be provided with relevant knowledge on health and risk factors concerning the populations they are dealing with. Health registries should record and monitor migrant health to facilitate migrant health research. (Table 3)

If a similar analysis is conducted on only the three most important principles in each country, instead of ten, *accessibility *features in eleven countries, *culturally sensitive care *in ten, *communication *in nine and *empowerment *in eight. All the remaining priorities featured among the three most important ones, but in less than half of the countries.

### Discordance

Discordance within and between countries concerning factors experts considered to be important was not infrequent. Of the 120 round 2 scores containing discordant scores, 99 (83%) were voiced by 13 of the 126 experts who completed the Delphi process in 7 of the 16 countries. None of the four expert categories (academia, NGO, policy makers, practitioners, and no professional category) were over-represented in these discordant voices. Final factors containing discordant scores were frequent in: Greece (10/12), Austria (7/11), UK (7/12) and Portugal (7/13). However, the discordance in these final factors was all due to one single expert in each country, with the exception of the UK with 2 discordant experts. No other country had more than 3 final factors containing discordant scores.

The whole question of migrant-specific health care is seen as a false problem by discordant experts in Belgium ("the key difference is socioeconomic"), Finland ("everyone is culturally different, not just migrants") and France ("pinpointing cultural needs creates health care ghettos"). Within country discordance, as defined in the Methods section above, remained with respect to 15 good practice factors in seven countries (Table 4). In Austria and Belgium, discordance even remained in the final list of ten most important factors. In Austria, there was disagreement on the need for employing staff speaking migrant languages, translated information material, and measures directed at preventing and diminishing discrimination. In Belgium, discordance persisted on the importance of providing specific information about health insurance and other financial support measures. In Lithuania, experts disagreed about the need for providing information about the health care system. In France and Poland, experts disagreed about the need for specific epidemiological information on areas with high migrant populations. French experts also disagreed about the need for having experts providing extensive training to health care professionals to be aware of ethnic and cultural issues, with opponents considering this to be a trap, arguing that the most important principles for health professionals are to take sufficient time and have access to an interpreter.

**Table 4:** Consensus and discordance concerning factors of good practice in health care for migrants in Europe.

Country	Factors presented at 2^nd ^round (n)	Consensus^1^ 2^nd ^round	Consensus final (3^rd^) round	Factors containing discordance in final list^2^
AUSTRIA	17	12%	29%	4
BELGIUM	21	0%	38%	2
DENMARK	32	31%	75%	0
ENGLAND	28	11%	71%	0
FINLAND	22	9%	64%	0
FRANCE	40	12%	61%	2
GERMANY	30	3%	43%	3
GREECE	24	17%	71%	0
HUNGARY	16	6%	81%	0
ITALY	11	9%	82%	0
LITHUANIA	35	14%	49%	1
NETHERLANDS	26	4%	81%	1
POLAND	31	3%	61%	2
PORTUGAL	18	6%	22%	0
SPAIN	14	-^3^	80%	0
SWEDEN	30	20%	83%	0

^1 ^Consensus: Percentage of experts' scores that are 1 point or less from the rounded average score for each factor

^2 ^Discordance: Number of scores that are 3 points or more from the rounded average score

^3 ^Spain had two rounds only.

German experts disagreed on the need for improving contextual societal factors, such as the social acceptance and social support of migrants as ways of improving accessibility and quality of health care. The presence of a discordant voice generally resulted in that factor having a significantly lower final average score. This was the case, for example, in Germany for the factor concerning the need for health care providers to reflect on their own cultural backgrounds. In the Netherlands, experts had highly opposing views on the need for customized care for migrants, even on a small-scale, action-specific and temporary basis. In Portugal, promoting positive attitudes towards migrants and fighting against discrimination in health care professionals was excluded from the final national good practice list due to two discordant votes (both from academics).

Discordance also existed between the 16 participating countries. For example, coherent national, regional and local governance was seen as an advantage in Belgium and as a disadvantage in the UK, where political correctness in health care policy might have created resistance and discord.

## Discussion

Sixteen European countries, with varying migration histories, participated in this research. The national consensus lists of principles of good care fell into nine thematic categories for which there was a considerable level of consensus across countries. Four out of the nine principles figured in the final lists of 12 countries, and eight in the final lists of at least eight of the participating countries. Best practice should guarantee easy access to health care resources and equal rights. Migrants should be empowered to make optimum use of culturally sensitive health care services. Good practice means quality individual care, provided when needed and adapted to migrants' needs in terms of: communication, attitudes, empathy and non-discrimination.

Organising a Delphi process with experts in 16 different countries is a challenge. The study did not to set out to identify consensus across all 16 countries, but chose to identify consensus lists at national levels. This approach was considered more useful given the different contexts of the national health systems. It also facilitated participation, in that experts were able to report in their own language, resulting in more detailed explanations regarding the suggested factors.

A limitation of this study is the representativeness of the experts participating in each country. Although the project set out to recruit experts from different professional backgrounds, medical doctors and professionals working in the field of mental health were over-represented in some countries. Furthermore, experts in several countries were all based in the capital city, with possible implications for the generalisability of findings to more rural contexts. The presence of experts with strong discordant views on particular factors had an important effect on the outcome of the Delphi process. Furthermore, in countries with limited immigration experience, discussion about the need for culturally sensitive health care was more limited. In the current analysis, these countries nonetheless received the same weight as countries with greater experience in providing health care to large migrant populations.

Differences between countries' health care systems and social contexts are likely to have influenced the selection of factors. The development and implementation of migrant health policies is a challenging task for governments, given the highly political nature of public policy on immigration in most European countries today [23]. Recent discourse in Europe on the necessity of integrating immigrants, and the resulting debate on what host societies should expect from immigrants through this integration process, may well have impacted on the Delphi process. As researchers reported, issues that were highly debated within individual countries during the time of data collection, inevitably appeared on the initial lists of best practice factors, even though they often did not reach consensus during the Delphi process. Other practices might have been so accepted and widespread in certain countries that they were taken for granted even by the experts, did not end up as priorities. For example, formal interpreting services are readily available in the Netherlands at many levels of the health care system, although not necessarily in every single situation in which they might be useful. Recently the government decided to discontinue financing interpreter services for health care from 2012 on [29]. In the case of Germany, there is an on-going debate within the mental health sector about the advantages and disadvantages of therapists with the same ethnic background as the patients compared to therapists using trained interpreters [30]. Also the pros and cons of specialized services compared to culturally sensitive standard care are subject of public debate in several countries. In Austria, the current debate is less adapting the health care system to population diversity, but stronger about immigrants having to gain German language skills and integrating into the labour market and therefore contribute to the health care system [31].

The widespread debate on the integration of immigrants into European societies may impact on the provision of language support and translated information material for migrant patients. This might be a reason for why no principle on migrant empowerment was agreed in Denmark [32], where in 2011 the government has introduced a fee on interpreter services for migrants having resided in the country for more than seven years [33]. In Belgium, immigration contexts and political views concerning immigration in a broad sense, reception of asylum seekers and regularisation of undocumented migrants vary between regions and regional political trends, thus limiting the chance to reach overarching political decisions [34]. In Poland, which has a short history of immigration [32], the debate is not about immigration itself, but more about the quality of health care and its accessibility for immigrants and especially about services for asylum seekers. Although Greece is currently facing a large influx of immigrants, cultural mediators and other facilities are still not available as the challenge of immigration has still to be addressed [32,35].

Given these greatly varying national realities, it could be seen as surprising that such a broad European consensus exists on the need for health care services to take ethnic cultural diversity into account. The right to equal access to health care for immigrants, without barriers, is regarded as a priority in all participating countries. A large majority of experts agree that empowering immigrants regarding health and health care, not the least by improving communication and developing culturally sensitive health care services, will facilitate access to and quality of care. An attitude of respect, trust and openness without discrimination forms the basis around which all other best practice principles and activities can develop.

## Conclusion

The identified principles for migrant sensitive and accessible health care are not new. However, the results of this study underline the current broad consensus in Europe among experts coming from different backgrounds.

Several of the identified principles are also reflected in recent policy documents such as The Amsterdam Declaration: towards migrant friendly hospitals in an ethno-culturally diverse Europe [36]; the United Kingdom Race Relations Act [25]; frameworks developed under the Portugal Presidency of the Council of the European Union and the Council of Europe [20,21]; and the WHO's Global Consultation on Migrant Health which took place in Spain in 2010 [1,25]. It may well be time to develop a European Charter on the right to, and the need for accessible, culturally sensitive health care for all citizens in Europe, wherever their origins. Through the Charter governments should commit themselves to creating and supporting all necessary conditions for the development of culturally sensitive health care. Although, more research is needed to develop the views of stakeholders on this question, the present consensus list of principles of best practice may be, in our opinion, a significant step in this direction.

## Competing interests

The authors declare that they have no competing interests.

## Authors' contributions

WD contributed to the study design, was in charge of study implementation and data analysis, and drafted the manuscript. SP designed the study protocol, was in charge of the overall management of the study and helped draft the manuscript. TG participated in data analysis and helped draft the manuscript. MB supported study co-ordination. All authors collected national data and are guarantors of the data in their country; they commented on successive drafts of the manuscript and approved its final version.

## Pre-publication history

The pre-publication history for this paper can be accessed here:

http://www.biomedcentral.com/1471-2458/11/699/prepub
